# Misdiagnosis and Clinical Insights into Acral Amelanotic Melanoma—A Systematic Review

**DOI:** 10.3390/jpm14050518

**Published:** 2024-05-13

**Authors:** Fortunato Cassalia, Andrea Danese, Enrico Cocchi, Elisabetta Danese, Francesca Ambrogio, Gerardo Cazzato, Marcodomenico Mazza, Anna Zambello, Anna Belloni Fortina, Davide Melandri

**Affiliations:** 1 Unit of Dermatology, Department of Medicine, University of Padova, 35131 Padua, Italy; anna.zambello@studenti.unipd.it (A.Z.); anna.bellonifortina@gmail.com (A.B.F.); 2 Unit of Dermatology, Department of Medicine, University of Verona, 37134 Verona, Italy; andrea.danese_02@studenti.univr.it (A.D.); elisabetta.danese_02@studenti.univr.it (E.D.); 3 Department of Medical and Surgical Sciences, University of Bologna, 40126 Bologna, Italy; enrico.cocchi3@unibo.it (E.C.); davide.melandri2@unibo.it (D.M.); 4 Department of Precision Medicine and Genomics, Department of Medicine, Columbia University, New York, NY 10027, USA; 5 Neonatal and Pediatric Intensive Care Unit, AUSL Romagna, 47121 Forlì, Italy; 6 Section of Dermatology and Venereology, Department of Precision and Regenerative Medicine and Ionian Area (DiMePRe-J), University of Bari “Aldo Moro”, 70124 Bari, Italy; francesca.ambrogio@policlinico.ba.it; 7 Section of Molecular Pathology, Department of Precision and Regenerative Medicine and Ionian Area (DiMePRe-J), University of Bari “Aldo Moro”, 70124 Bari, Italy; gerardo.cazzato@uniba.it; 8 Soft-Tissue, Peritoneum and Melanoma Surgical Oncology Unit, Veneto Institute of Oncology IOV-IRCCS, 35128 Padova, Italy; marcodomenico.mazza@iov.veneto.it; 9 Regional Center for Pediatric Dermatology, Department of Women’s, and Children’s Health (SDB), University of Padua, 35121 Padua, Italy; 10 Cesena Skin Clinic and Regional Skin Bank, AUSL Romagna, 47121 Forlì, Italy

**Keywords:** acral melanoma, amelanotic melanoma, acral amelanotic melanoma, melanoma, misdiagnosis, sarcoma, melanoma mimicking, acral skin lesions

## Abstract

Background: Acral amelanotic melanomas (AAMs), a rare subset of melanomas located on acral sites such as the palms, soles, and subungual areas, are diagnostically challenging due to their lack of typical pigmentation and often benign clinical appearance. Misdiagnosis is common, leading to delays in treatment and potentially worse outcomes. This systematic review aims to synthesise evidence on cases of AAM initially misdiagnosed as other conditions, to better understand their clinical and epidemiological characteristics, diagnostic pitfalls, and management strategies. Methods: A comprehensive search of the MEDLINE/PubMed, EMBASE, and SCOPUS databases was conducted up to March 2024. Case reports and small case series of AAMs initially misdiagnosed as other conditions were included. Data on patient demographics, clinical presentation, and diagnostic methods were collected and analyzed. Results: Of the 152 records identified, 26 cases from 23 articles met the inclusion criteria. A demographic analysis revealed that the gender distribution appears to be perfectly balanced, with an age range of 38 to 91 years. Misdiagnoses included non-healing ulcers or traumatic lesions (37.5%), benign proliferative lesions (29.2%) and infectious lesions (20.8%). The foot was the most affected site (53.8%). Notably, a histological evaluation was performed in 50% of cases involving the upper extremities, in contrast to only 7.1% of cases involving the foot and 0% of cases of the heel. This discrepancy suggests a reluctance to perform biopsies in the lower extremities, which may contribute to a higher misdiagnosis rate in these areas. Conclusions: The underutilization of biopsy in the diagnosis of lower extremity lesions contributes significantly to the misdiagnosis and delay in treatment of AAMs. Especially when the clinical assessment and dermoscopy are inconclusive, biopsies of suspicious lesions are essential. Immunohistochemistry and markers such as PRAME are critical in differentiating melanoma from other malignancies such as clear cell sarcoma. This review highlights the need for increased vigilance and a proactive diagnostic approach to increase early detection rates and improve prognostic outcomes.

## 1. Introduction

Melanoma, a type of skin cancer originating from melanocytes, presents a wide array of clinical manifestations, often characterised by the appearance of pigmented lesions [1]. However, a subset of melanomas, known as acral amelanotic melanomas (AAMs), poses distinct challenges for clinicians and pathologists due to their atypical features [2]. Comprising only a small percentage (approximately 2–3%) of all melanoma cases, acral melanomas are located on the palms, soles, and subungual areas, making their diagnosis particularly complex [1,3]. Among acral melanomas, AAMs present an even greater diagnostic hurdle, as they lack the typical pigmentation associated with most melanomas. Consequently, these lesions can be mistakenly identified as benign conditions, leading to delayed diagnosis and appropriate treatment, with potentially severe consequences [1,2,4]. The aggressive nature of AAMs means that a delayed diagnosis can lead to missed opportunities for early intervention, significantly impacting patient outcomes. Adding to the diagnostic challenge is the anatomical location of acral melanomas, which frequently overlaps with various benign lesions like warts, calluses, and cysts [5,6,7]. These similarities can obscure a correct diagnosis and increase the risk of misidentifying the malignancy. Recognizing the importance of early detection and an accurate diagnosis, an emerging body of literature has documented cases of AAMs initially misdiagnosed as other lesions [5,8]. Despite the presence of case reports and small case series, a comprehensive and systematic review is required to synthesise the existing evidence and shed light on this diagnostic dilemma. Such a review can offer valuable insights into the clinical and epidemiological features of these disguised melanomas, aiding clinicians in making informed decisions when faced with suspicious lesions in acral regions. Thus, this systematic literature review aims to address the gaps in knowledge surrounding this topic.

## 2. Materials and Methods

### 2.1. Study Design

This systematic review aims to investigate cases of AAMs that were initially misdiagnosed as other lesions. The review follows the Preferred Reporting Items for Systematic Reviews and Meta-Analyses (PRISMA) guidelines [9]. Our study has not been registered with a protocol on PROSPERO.

### 2.2. Search Strategy

We conducted a comprehensive search of the relevant literature in the following electronic databases: MEDLINE/PubMed, EMBASE, and SCOPUS. The search was conducted up until March 2024. The PubMed search strategy was adapted as follows: “acral amelanotic melanoma”. Similar search strategies were applied to the other electronic sources. Duplicate records were removed, and four investigators (FC, AD, EC, and ED) independently screened the titles and abstracts of the retrieved records, excluding those not relevant to the review’s scope. The full text of potentially eligible records was assessed for inclusion criteria. Additionally, we hand-searched the reference lists of the included studies for further relevant articles. Studies not involving human subjects and studies in languages other than English were excluded.

### 2.3. Inclusion Criteria

The inclusion criteria for this systematic review were carefully constructed according to the PRISMA guidelines. Only case reports and small case series published in English that accurately described cases of acral amelanotic melanoma initially diagnosed as another condition were included. A key requirement was that each study was available in full to enable us to examine its details. We also considered the relevance of each study, screening titles and content to ensure relevance to our research topic.

### 2.4. Data Collection

Four investigators (FA, GC, MM, and AZ) independently extracted the relevant data from the included articles. The data extraction process included study characteristics, patient demographics, tumour information, and outcome measures. Two different investigators (ABF and DM) reviewed the extracted data to ensure accuracy, and any discrepancies were resolved through consensus.

### 2.5. Assessment of Study Quality

The quality of the included studies was assessed, based on eight criteria adapted from the Joanna Briggs Institute (JBI) critical appraisal tool [10]. The criteria included the following: (i) clear inclusion criteria for patients, (ii) valid methods for identifying the initial condition, (iii) valid methods for identifying the final condition, (iv) the consecutive inclusion of patients in case series, (v) clear reporting of demographics, (vi) clear reporting of clinical information, (vii) reporting of the time of the second assessment, and (viii) reporting the overall survival. Two investigators (EC and GC) independently assessed the risk of bias in the included studies, and any discrepancies were resolved through consensus among all the authors.

### 2.6. Data Synthesis

The study selection process was illustrated using a flowchart. Pertinent data extracted from the included studies were summarised in a tabular format. Due to the inclusion of case reports and small case series, conducting a meaningful meta-analysis was not feasible. Therefore, a narrative synthesis of the included studies was performed to present the findings.

### 2.7. Statistical Analysis

We conducted a comprehensive statistical analysis of the cases identified. Descriptive statistics were compiled to summarise patient demographics, clinical features, diagnostic methods, and treatment outcomes. Chi-squared and Mann–Whitney tests were applied to assess the association between the variables under study. All analyses were performed using R version 4.3.3.

### 2.8. Limitations

This systematic review has several limitations, including the small number of cases available, which may not fully represent the diversity and complexity of acral amelanotic melanoma. In addition, the potential under-reporting of cases in the literature due to misdiagnosis or non-recognition further complicates a comprehensive analysis. There is also a risk of publication bias, as cases that are successfully diagnosed are more likely to be reported. The retrospective nature of the case reports and series included may limit the robustness of the conclusions that can be drawn regarding diagnosis and treatment outcomes. Finally, this review lacks a comparative analysis with cases that were correctly diagnosed at baseline, which limits an understanding of the diagnostic process and outcomes between misdiagnosed and correctly identified cases. This lack hinders our ability to draw definitive conclusions about the effectiveness of diagnostic strategies and the potential for improved patient management.

## 3. Results

### 3.1. Search Results

**Figure 1:**
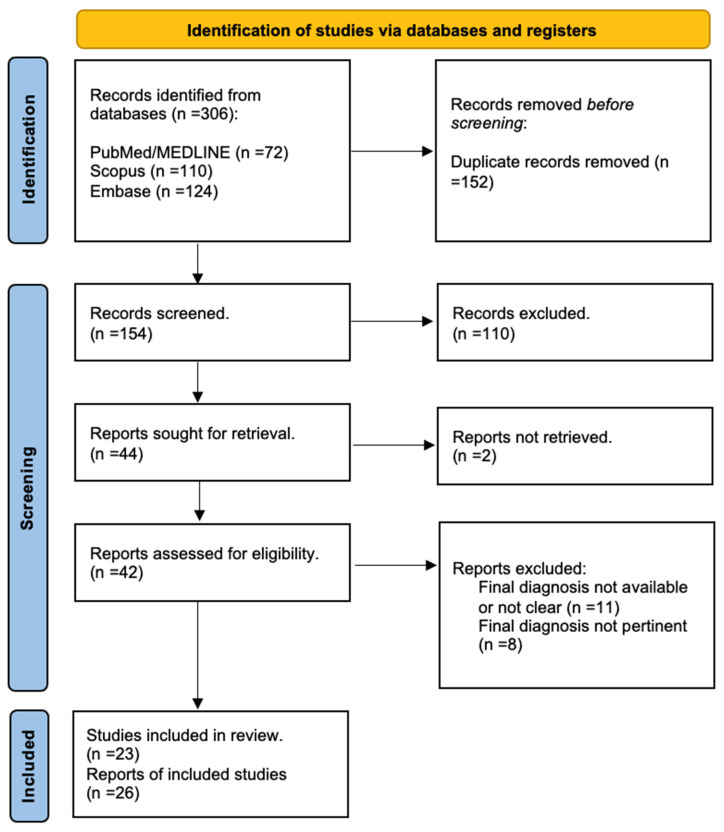
PRIMA flowchart.

A comprehensive search of the main databases yielded 152 unduplicated records. We excluded 110 records based on the title or abstract and identified 42 potentially eligible records. At this stage, 11 records were excluded due to an unreported or unclear final diagnosis, 8 records were excluded due to an irrelevant final diagnosis, and 2 records were excluded due to an unavailable or unsatisfactory full text. In the end, 23 unique articles with a total of 26 clinical cases were included (Figure 1).

### 3.2. Narrative Synthesis of the Findings

**Figure 2:**
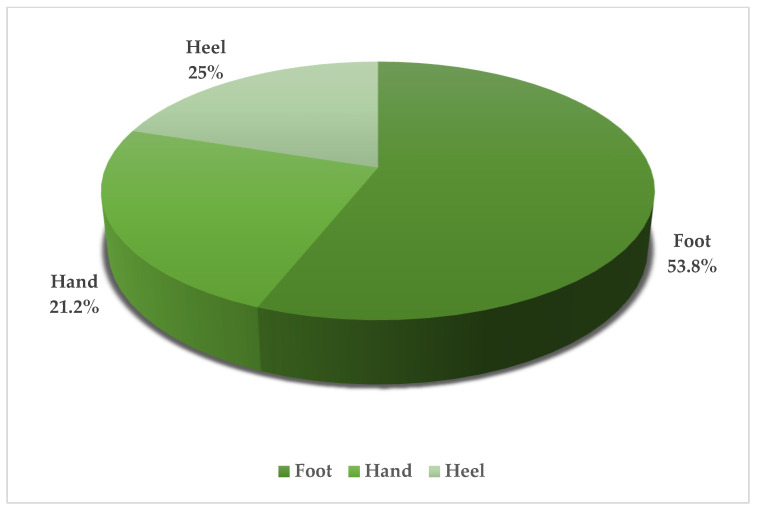
Anatomical Site.

**Figure 3:**
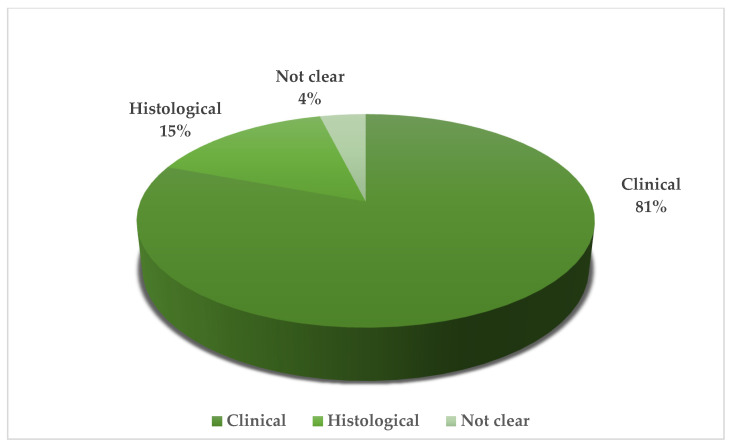
Type of initial diagnosis.

Twenty-seven patients diagnosed with acral amelanotic melanoma (AAM) were enrolled. Patients ranged in age from 38 to 91 years, indicating that AAM is not restricted to a specific age group. The gender distribution appears to be perfectly balanced, with 50% male and 50% female. The initial misdiagnosis was non-healing ulcers or traumatic injury in 37.5% of cases. This was followed by misclassification as benign proliferative lesions and infectious lesions in 29.2% and 20.8% of cases, respectively. There was also a significant proportion of cases (12.5%) that were initially misdiagnosed as other malignant tumors. The most common site of these lesions is the foot, accounting for 53.8% of cases. This is followed by the heel (25%) and hand with 21.2%. (Table 1, Figure 2) There is a greater propensity to use histology for hand injuries, with a histological evaluation performed in 50% of such cases (Table 2); in contrast, lesions on the foot and heel underwent histological analysis in only 7.1% and 0% of cases, respectively, highlighting a potential oversight in the assessment process for lower extremity lesions. (Figure 3) Treatment approaches were varied but predominantly surgical. Most patients underwent surgery alone. A significant proportion required additional interventions. Less common treatments included isolated limb perfusion, chemotherapy, and immunotherapy, demonstrating the aggressive nature of the disease and the variety of therapeutic strategies employed. Immunohistochemical analysis could play a key role in diagnosis, although unfortunately not all reported cases included an immunohistochemistry. These markers were critical in confirming the melanocytic origin of the tumour cells in the absence of melanin pigmentation, which is typically a hallmark of melanoma. This review highlights a critical gap in the initial assessment based on lesion location. Lesions on the hands were more likely to undergo an immediate histological examination, whereas examination of those on the foot or heel was often delayed, raising concerns about the potential underdiagnosis or delayed diagnosis of lesions on the lower extremities. This finding highlights the need for a high index of suspicion and a comprehensive diagnostic approach for acral lesions, particularly in the lower extremities, due to the high potential for initial misdiagnosis and the consequent impact on timely and effective management of AAMs.

### 3.3. Clinical Presentation

In clinical practice, commonly misdiagnosed lesions present with a wide range of appearances and symptoms. They range from apparently benign and asymptomatic pink nodules to more alarming manifestations, such as ulcerated and painful lesions commonly observed on acral sites such as the heels or fingertips. Among these, lesions with hyperkeratotic features are particularly misleading, often resembling benign conditions such as viral warts or corns, thus complicating the diagnostic process. On the other hand, the lack of pigmentation and the diversity of presentation and location of these lesions add significantly to the diagnostic challenges. For example, simple nodules or hyperkeratotic plaques may appear harmless, and misidentifications lead to delays in diagnosis and appropriate intervention.

## 4. Discussion

Acral amelanotic melanoma (AAM) represents a major diagnostic challenge due to its rarity, aggressive behavior, and lack of melanin pigmentation typical of most melanomas [3,31]. The propensity of this disease to affect acral sites such as palms, soles, or subungual areas and its lack of staining can lead to these lesions being mistaken for benign conditions such as plantar warts, ulcers, or fungal infections, highlighting the critical need for greater clinical awareness [1,4,25]. Because of their non-pigmented and often harmless appearance, patients with AAMs may delay in seeking medical attention for ulcers or nodules that do not heal [3]. Dermoscopically, an AAM has several features that distinguish it from pigmented melanoma. AAMs may present with irregular vascular patterns, including polymorphous vessels and areas of milky red erythema, which may facilitate dermoscopic identification [5,32,33]. Histologically, AAM is characterised by atypical melanocytes lacking melanin [32]. This characteristic makes diagnosis difficult, especially for those who do not specialise in melanoma. An immunohistochemistry with markers such as S100, HMB-45, Melan-A, SOX-10 Ki-67, and PRAME can be very helpful in confirming the melanocytic origin of the lesion and narrowing the diagnostic suspects [8,34,35]. On the other hand, it is important to underscore that, while S-100, Melan-A, and HMB-45 are very useful in confirming a diagnosis of melanoma, SOX-10 is the most reliable immunohistochemical marker in detecting the neuro-ectodermal origin of a specific lesion and concluding a diagnosis with great confidence. Furthermore, confocal reflectance microscopy (RCM) provides a non-invasive means of observing cellular and subcellular structures of the skin in vivo with near-histological resolution. In AAM, RCM can identify atypical melanocytes at the dermo-epidermal junction and within the epidermis, even in the absence of pigmentation [36,37]. In addition to benign conditions that may mimic AAM, it is important to remember that there are some very aggressive neoplasms with AAM-like features [14,20]. For example, an important differential diagnosis is that between AAM and acral lentiginous melanoma (ALM), given their acral localization but different clinical presentation. The lack of pigmentation in AAM requires the diagnosis to be based on the lesion’s texture, growth pattern, and non-pigmented dermoscopic features [12,18]. In contrast, the presentation of ALM, often characterised by brown or black shades in a striated or diffuse pattern, is more likely to raise suspicion of melanoma [8,38,39]. Dermoscopy is very helpful in differentiating AAM from ALM; while the diagnosis of AAM may be based on subtle vascular patterns and the absence of pigmented structures, ALM may present with more definitive indicators of melanoma, such as pigment networks [5,8]. Another important aspect is the differentiation of AAM from other malignancies such as sarcomas, which may confuse the physician due to their tendency to involve similar acral sites [40,41]. The differentiation of AAM from sarcomas requires careful clinical evaluation, supported by histological and immunohistochemical analysis [40,42]. In this case, the use of PRAME as an immunohistochemical marker could be a valuable diagnostic aid; indeed, PRAME, which is expressed in melanoma but not in most sarcomas, could be an important discriminatory tool, improving the diagnostic accuracy of AAM [40,41,42,43,44]. Alternatively, traditional genetic studies may be very useful in the differential diagnosis between these two neoplasms [40,42]. Because of its mimetic appearance and difficult clinical interpretation, the diagnosis of AAM can sometimes come late in the patient’s diagnostic course, sometimes even years later; in this regard, it should be noted that the statistical analysis within the systematic review reveals a remarkable finding: the initial diagnostic approach seems to be significantly influenced by the anatomical location of the lesion (p: 0.05) [31]. There is a greater propensity to use histology for hand injuries, with histological evaluation performed in 50% of cases (Table 2). In contrast, foot or heel injuries are much less likely to be submitted for histological examination at presentation, with only 7.1% and 0% of cases, respectively, receiving such an analysis. These data highlight a significant trend in clinical practice that deserves attention. Lower limb injuries, particularly acral regions of the foot or heel, may not receive the necessary level of investigation. These findings suggest a potential underestimation of the severity of acral injuries of the lower extremity, which are often dismissed as benign and treated less aggressively in terms of diagnostic confirmation [30]. Given the aggressive nature and diagnostic challenges of AAM, this statistic highlights the urgent need for a change in clinical vigilance. It suggests that healthcare professionals should approach acral lesions of the lower limbs with a higher index of suspicion and more readily consider histological evaluation. Since AAM can be masked by benign lesions, especially in the early stages, a more meticulous and timely approach may lead to earlier diagnosis and intervention, which are critical in the management of this potentially life-threatening condition [5].

## 5. Limitations

This systematic review has highlighted the significant diagnostic challenges posed by AAM, but it is important to acknowledge the inherent limitations of our study, which may affect the generalizability and robustness of our conclusions. First and foremost, the relatively small number of cases included in the review may limit the ability to fully represent the diversity and complexity of AAM presentations. This small sample size reflects not only the rarity of the condition but also potential under-reporting in the literature, due to misdiagnosis or non-recognition of the condition. In addition, the studies included are predominantly case reports and case series, which are inherently at a high risk of bias, including publication bias. Cases that are successfully diagnosed and reported are more likely to represent atypical or severe presentations, which may not accurately reflect the wider spectrum of disease presentations. This bias could distort the perceived severity and outcomes of the disease. The retrospective nature of the case reports and case series included also limits the strength of the conclusions we can draw about diagnostic methods and treatment outcomes. Retrospective studies are more susceptible to missing data and recall bias, which can make it difficult to interpret the results. Another limitation is the lack of a comparative analysis with cases that were correctly diagnosed at baseline. This omission limits our understanding of the diagnostic process and outcomes between misdiagnosed and correctly identified cases and hinders our ability to draw definitive conclusions about the effectiveness of current diagnostic strategies and the potential for improved patient management. Furthermore, our findings are limited by the variable quality and detail of case reports, with inconsistent reporting of key clinical details such as histopathological findings and long-term outcomes. This inconsistency makes it difficult to perform a rigorous comparative analysis. Considering the limitations, the conclusions drawn from this review are interesting and open wide avenues for research but should be interpreted with caution. Future research should focus on larger, prospective studies to gain a more complete understanding of the disease and to validate the findings reported here.

## 6. Conclusions

This systematic review presents a statistically significant finding that the initial diagnostic approach to AAM is strongly influenced by the anatomical location of the lesion. Lesions on the hands are more likely to undergo immediate histological evaluation than those on the foot or heel. This discrepancy highlights a significant trend in clinical practice that deserves attention. Acral lesions, particularly in the lower extremities, often do not receive the necessary level of investigation, potentially delaying appropriate diagnosis and treatment. Due to the aggressive nature and diagnostic challenges associated with AAM, this finding highlights the urgent need for increased clinical vigilance and a more systematic approach to the histological evaluation of suspicious lesions in these regions [45,46,47]. For effective clinical practice and to minimise misdiagnosis, the biopsy of suspicious acral lesions on the lower limbs is essential, especially when the clinical assessment and dermoscopy are inconclusive. Clinicians should be alert to lesions that do not resolve, such as warts and ulcers, as these should prompt consideration of more serious conditions. Immunohistochemistry and the use of markers such as PRAME can be very helpful in distinguishing melanoma from other malignancies such as clear cell sarcoma [41,44]. However, despite the interesting results, the conclusions of this review must be tempered by the limitations discussed above. In the future, it is important to promote a greater awareness of and education about AAM among healthcare professionals. This may lead to earlier diagnosis and intervention, which are essential to improve outcomes for patients with this elusive and dangerous form of melanoma. Finally, a multidisciplinary approach involving dermatology, pathology, and oncology is essential to improve the diagnosis and management of AAM. The main challenge in diagnosing AAM is to recognise the malignant potential of the lesions from the earliest clinical assessments, taking advantage of available diagnostic tools and promoting a multidisciplinary model of care.

## Figures and Tables

**Table 1:** Case report and case series included.

Authors	Year	Sex	Age	Site	First Diagnosis	Final Diagnosis	Breslow	Mitotic Rate	IHC	Stage	Therapy	OS	Ref
Deng, W. et al.	2019	F	42	Foot	Infectious lesions	AAM	2 mm	>1/mm^2^	S100, HMB45, and Melan-A, Ki67	T2aN1bM0	Surgery plus lymph node dissection	/	[2]
Cantwell, P. et al.	2019	M	70	Foot	Non-healing ulcers/traumatic lesions	AAM	4.3 mm	/	/	/	Surgery	/	[3]
Shawa, H. J. et al.	2022	M	67	Heel	Infectious lesions	AAM	1.5 mm	/	/	pT2b	Surgery	9 months	[5]
Bouceiro-Mendes, R. et al.	2021	F	66	Heel	Non-healing ulcers/traumatic lesions	AAM	6 mm	/	/	/	Surgery, ILP	/	[11]
Koblinski, J. E. et al.	2022	F	58	Heel	Benign proliferative lesions	AAM	/	/	/	/	/	24 months	[12]
Rachadi, H. et al.	2016	M	68	Heel	Benign proliferative lesions	AAM	2 mm	/	C-kit neg	/	Surgery, CT		[13]
Zhang, J. et al.	2022	M	61	Foot	Non-healing ulcers/traumatic lesions	AAM	/	/	Ki67, Melan-A, HMB-45	pT4b	Surgery	/	[14]
Cozzani, E. et al.	2019	F	80	Foot	Benign proliferative lesions	AAM	0.4 mm	/	S100, Melan-A, and HMB-45	/	/	/	[15]
Gencoglan, G. et al.	2011	M	68	Hand	Infectious lesions	AAM	>4 mm	/	S100, HMB-45, vimentin pos	/	Chemotherapy	/	[16]
Mohammed Saeed, D. et al.	2019	F	83	Heel	Non-healing ulcers/traumatic lesions	AAM	10 mm	9/mm^2^	Melan A	pT4bN0	Surgery	/	[17]
Okhovat, J. P. et al.	2019	M	50	Foot	Infectious lesions	AAM	8.3 mm	5/mm^2^	/	pT4aN3	Surgery, INF alpha, immunotherapy	36 months	[18]
Hara, M. et al.	1993	F	74	Hand	Benign proliferative lesions	AAM	/	/	S100	/	Surgery	/	[19]
		M	61	Hand	Benign proliferative lesions	AAM	/	/	S100	/	Surgery	/	[19]
		M	47	Hand	Benign proliferative lesions	AAM	/	/	S100	/	Surgery	/	[19]
Yasuoka, N. et al.	1999	M	70	Foot	Non-healing ulcers/traumatic lesions	AAM	2.5 mm	/	S100, HMB-45	/	Surgery	/	[20]
Mendes, M. S. et al.	2013	F	38	Foot	Unclear	AAM	>3 mm	/	/	/	Surgery, lymph node dissection RT and adjuvant treatment with interferon	/	[21]
Matusiak, L. et al.	2008	F	74	Foot	Other malignancies	AAM	9 mm	<1/mm^2^	Melan A pos; negative EMA and Ck-19 negative	pT4	Surgery		[22]
Ghariani, N. et al.	2008	M	86	Hand	Non-healing ulcers/traumatic lesions	AAM	/	/	HMB-45, melan A	/	Surgery	8 months	[23]
Kato, T. et al.	1997	F	59	Foot	Other malignancies	AAM	/	/	S100, HMB-45	pT4b	Surgery	15 months	[24]
		F	62	Heel	Other malignancies	AAM	/	/	/	/	Surgery	60 months	[24]
Hussin, P. et al.	2012	M	80	Foot	Non-healing ulcers/traumatic lesions	AAM	/	/	/	/	Surgery	12 months	[25]
Sundell J.	2010	F	90	Foot	Non-healing ulcers/traumatic lesions	AAM	/	/	/	/	Surgery	/	[26]
Kutlu Ö. et al.	2016	M	91	Foot	Benign proliferative lesions	AAM	/	/	Melan-A, S100, HMB45	/	Surgery	/	[27]
Guilherme, M.R. et al.	2022	M	74	Foot	Non-healing ulcers/traumatic lesions	AAM	8.6 mm	Not reported	MELAN-A, HMB-45	/	Surgery	7 months	[28]
Karaja S.A. et al.	2024	F	39	Hand	Paronychia/infectious lesions/cutaneous leishmaniasis	AAM	/	/	/	/	Surgery/sentinel lymph node biopsy		[29]
De Giorgi V. et al.	2006	F	53	Foot	Infectious lesions	AAM	>6 mm	/	/	/	Surgery/lymph node excised and adjuvant treatment with interferon	12 months	[30]

**Table 2:** Type of initial diagnosis.

Authors	Year	Sex	Age	Site	Type of Initial Diagnosis	Ref
Deng, W. et al.	2019	F	42	Foot	Clinical	[2]
Cantwell, P. et al.	2019	M	70	Foot	Clinical	[3]
Shawa, H. J. et al.	2022	M	67	Heel	Clinical	[5]
Bouceiro-Mendes, R. et al.	2021	F	66	Heel	Clinical	[11]
Koblinski, J. E. et al.	2022	F	58	Heel	Clinical	[12]
Rachadi, H., et al.	2016	M	68	Heel	Clinical	[13]
Zhang, J. et al.	2022	M	61	Foot	Clinical	[14]
Cozzani, E. et al.	2019	F	80	Foot	Clinical	[15]
Gencoglan, G. et al.	2011	M	68	Hand	Clinical	[16]
Mohammed Saeed, D. et al.	2019	F	83	Heel	Clinical	[17]
Okhovat, J. P. et al.	2019	M	50	Foot	Clinical	[18]
Hara, M. et al.	1993	F	74	Hand	Histological	[19]
		M	61	Hand	Histological	[19]
		M	47	Hand	Histological	[19]
Yasuoka, N. et al.	1999	M	70	Foot	Clinical	[20]
Mendes, M. S. et al.	2013	F	38	Foot	Clinical	[21]
Matusiak, L. et al.	2008	F	74	Foot	Not clear	[22]
Ghariani, N. et al.	2008	M	86	Hand	Clinical	[23]
Kato, T. et al.	1997	F	59	Foot	Histological	[24]
		F	62	Heel	Clinical	[24]
Hussin, P. et al.	2012	M	80	Foot	Clinical	[25]
Sundell J.	2010	F	90	Foot	Clinical	[26]
Kutlu Ö. et al.	2016	M	91	Foot	Clinical	[27]
Guilherme, M.R. et al.	2022	M	74	Foot	Clinical	[28]
Karaja, S.A. et al.	2024	F	39	Hand	Clinical	[29]
De Giorgi, V. et al.	2006	F	53	Foot	Clinical	[30]

## Data Availability

The data presented in this study are available on request from the corresponding author.
